# A useful tool for the safe diagnosis and control of the two main pandemics of the XXI century: COVID-19 and African Swine Fever disease

**DOI:** 10.1371/journal.pone.0282632

**Published:** 2023-03-06

**Authors:** Sandra Barroso-Arévalo, Marta Díaz-Frutos, Aleksandra Kosowska, Marta Pérez-Sancho, Lucas Domínguez, José Manuel Sánchez-Vizcaíno

**Affiliations:** 1 VISAVET Health Surveillance Center, Complutense University of Madrid, Madrid, Spain; 2 Department of Animal Health, Faculty of Veterinary, Complutense University of Madrid, Madrid, Spain; Plum Island Animal Disease Center, UNITED STATES

## Abstract

The COVID-19 pandemic and the disease triggered by the African Swine Fever virus are currently two of the main problems regarding public and animal health, respectively. Although vaccination seems to be the ideal tool for controlling these diseases, it has several limitations. Therefore, early detection of the pathogen is critical in order to apply preventive and control measures. Real-time PCR is the main technique used for the detection of both viruses, which requires previous processing of the infectious material. If the potentially infected sample is inactivated at the time of sampling, the diagnosis will be accelerated, impacting positively on the diagnosis and control of the disease. Here, we evaluated the inactivation and preservation properties of a new surfactant liquid for non-invasive and environmental sampling of both viruses. Our results demonstrated that the surfactant liquid effectively inactivates SARS-CoV-2 and African Swine Fever virus in only five minutes, and allows for the preservation of the genetic material for long periods even at high temperatures such as 37°C. Hence, this methodology is a safe and useful tool for recovering SARS-CoV-2 and African Swine Fever virus RNA/DNA from different surfaces and skins, which has significant applied relevance in the surveillance of both diseases.

## Introduction

In the last years, the world has been marked by two important health problems of viral origin affecting both public and animal health: the Coronavirus Disease 2019 (COVID-19) pandemic [1] and the re-emerging of African Swine Fever (ASF) disease [2].

COVID-19 is an infectious disease produced by the SARS-CoV-2 virus, which causes symptoms such as cough, fever, severe pneumonia, and death [3]. The virus was first detected in China at the end of 2019 and subsequently spread over the world, being declared a global pandemic by March 2020 [1]. The causative agent is a spherical virus of 100–160 nm in diameter, with an envelope containing single-stranded RNA (ssRNA) of positive polarity between 26 and 32 kilobases in length, belonging to the *Coronaviridae* family. The genome of the SARS-CoV-2 virus encodes four structural proteins: protein S (spike protein), protein E (envelope), protein M (membrane), and protein N (nucleocapsid) [4]. On the other hand, ASF is a fatal disease that affects susceptible animals from the *Suidae* family whose notification to the World Organization for Animal Health (WOAH) is compulsory [5]. It is, currently, the major concern for the pig industry, due to its socio-economic impact and the high lethality rate of the infection (almost 100% in the case of virulent isolates). The infection is caused by a large double-stranded DNA virus, the only member of the *Asfarviridae* family. The virus genome varies between 170 and 193 kbp and codes for between 150 and 167 proteins, including those required for virus replication [2, 6, 7].

Both diseases are, therefore, extremely important in terms of One Health. While the COVID-19 pandemic has highlighted the interdependence between animal and human infections [8], ASF disease, which is not a zoonotic disease, has evidenced the necessity of a global approach to controlling infectious diseases [9]. In both cases, an early and reliable diagnostic is critical for disease control. Although vaccination has proved to be an effective tool for limiting SARS-CoV-2 fatal outcomes [10], the spread of this virus is not still controlled since transmission can occur in vaccinated people [11]. The situation is even worse in the case of ASF disease since no vaccine is available for all the potential hosts yet [12]. These facts have promoted that control of both diseases is based on early detection, making critical the availability of control programs in which diagnosis can be made easily and rapidly in the hosts but also in the environment [13]. In this sense, environmental detection of both agents is crucial to identify potential sources of infection or evaluate if the virus is presented in a specific place or even in the animals’ skin [14–16]. For example, the detection of SARS-CoV-2 in surfaces from a hospital can reflect inadequate cleaning measures [17]. By comparison, a positive result for the ASF virus from an environmental sample from a pig farm may evidence its circulation among the housed animals without the need of testing them [14, 18].

Taking into account the risk of managing potentially infected samples with both viruses, biosecurity is a key point in sample processing. In this sense, methodologies that allow for pathogen detection with high rates of sensitivity and specificity and, at the same time, capable of inactivating the agent, are greatly needed. The main diagnosis technique for both viruses is the Polymerase Chain Reaction (PCR) [19, 20], which is a fast and very sensible molecular procedure based on the amplification of the genetic material. This technique requires the previous extraction of the genetic material of the virus. Once this step is conducted, the sample is not infectious anymore. However, the preceding steps are hazardous and should be conducted under high-level biosecurity conditions [21]. This aspect slows down the process, is time-consuming, and limits the number of laboratories trained to manage potentially infected samples. Thus, these difficulties may be solved if the samples were inactivated at the moment of sampling, provided that sample inactivation still allows the correct preservation of the sample or even enhance its preservation.

In order to achieve this key objective of improving and speeding up the diagnosis of these two relevant diseases, we have evaluated a new and safe methodology for non-invasive and environmental sampling of both SARS-CoV-2 and ASF viruses that inactivates the samples and increase their preservation capacities. This methodology is based on Dry-Sponges (3 M, Madrid, Spain) pre-hydrated with a new surfactant liquid that inactivates viruses and bacteria and, at the same time, increases nucleic acid preservation. Here, we demonstrate that the surfactant liquid effectively inactivates both viruses and enables the conservation of the sample for long periods even under high-temperature conditions in an *in vitro* assay.

## Material and methods

### Surfactant liquid

The surfactant liquid used in these experiments consisted of two previously prepared solutions and is protected by the Spanish patent n° P2115ES00. This surfactant liquid has been previously used for environmental and animal skin sampling in the case of the following pathogens: ASF virus [14], SARS-CoV-2 [15, 16], *Mycobacterium tuberculosis* complex [22], and porcine brucellosis [23].

### Virus and cells

For the *in vitro* experiments, Vero E6 cells (ATCC, Manassas, Virginia) and porcine leukocytes were used. Leukocytes were harvested and cultured following the procedure described in Chapter 3.9.1 of the Manual of Diagnostic Test and Vaccines for Terrestrial Animals 2022 Edition [24]. SARS-CoV-2 MAD6 isolated from a 69-year-old male patient in Madrid (Spain) was kindly provided by Dr. Luis Enjuanes from the National Biotechnology Centre at the Higher Council for Scientific Research. This virus was isolated with the patient’s consent and ethical approval from an ethics committee was in place for this viral strain to be isolated from the patient [25]. The Vero-adapted España70 (E70) and the hemoadsorbing genotype II Armenia07 ASFv isolates were obtained from the European Union ASF reference laboratory (Animal Health Research Center, Spanish National Research Council, CISA, INIA, Spain).

### Cell cytotoxicity evaluation of the surfactant liquid

To determine the cytotoxicity point in Vero E6 cells, the following protocol was performed. In a 96-well plate, serial dilutions of the surfactant liquid in cell medium using a 1:2 dilution factor were displayed, starting at 1:10 to 1:640 (50 μl/well). Every dilution was analyzed in triplicates. At the same time, a suspension of Vero E6 cells was added with a final concentration of 30,000 cells/well (50 μl/well). Three cell controls were also included in which 50 μl of the medium was added to achieve the final volume of 100 μl/well. The plate was then incubated at 37°C for 24 hours with an atmosphere of 5% CO_2_. After incubation, the cells were observed at microscopy to evaluate the presence of cytotoxicity and cell viability was determined using the MTT assay (Sigma-Aldrich, Missouri, US) [26]. The MTT assay is used to measure cellular metabolic activity as an indicator of cell viability, proliferation, and cytotoxicity. Briefly, the culture medium was removed from the plate and 100 μl of the MTT solution (1 mg/ml) was added to each well. The plate was incubated at 37°C for 3 hours with an atmosphere of 5% CO_2_. After incubation, formazan crystals were dissolved by adding an amount of Dimethyl sulfoxide equal to the original culture medium volume (DMSO, Sigma-Aldrich) in a gyratory shaker at 600 rpm. Then, the absorbance was determined at 595 nm using an Anthos 2001 plate reader (Labtec, Salzburg, Austria). Absorbance values of the wells at the different dilutions were compared with the absorbance in the control cell wells using the Mann-Whitney U (M-N U) test in SPSS software (IBM, Somar, NY, USA).

Cytotoxicity was assessed in leukocyte culture in the same way as described for Vero E6 cells with the following changes: cells were distributed in 96 well cell culture plates adding 300,000 cells/well and incubated at 37°C for 4 days with a 5% CO_2_ atmosphere allowing leukocytes to mature. After the incubation period, the cell medium was replaced with 100 μl/well of 1:2 serial dilutions of the surfactant liquid. As in the measurement of cytotoxicity in Vero E6 cells, the result was evaluated after 24 hours by observation at microscopy and by performing the MTT assay.

### Evaluation of virus inactivation properties of the surfactant liquid in vitro

For this objective, three similar *in vitro* experiments based on virus cell culture were conducted, one with the SARS-CoV-2 virus and the other two with the E70 and Arm07 ASF virus isolates. In the three cases, inoculums were used at an initial concentration of 10^6^TCID_50_/ml. To demonstrate the viral inactivation capacity of the surfactant liquid, a 1:2 dilution of the inoculum with the surfactant liquid was performed and incubated at 4°C for 5 minutes to allow viral inactivation. Then, the virus-surfactant liquid mixture was diluted with FBS-free cell medium to achieve a final dilution of 1:4. In this suspension, the final concentration of both viruses was 2.5x10^5^TCID_50_/ml, while the surfactant liquid was at a concentration of 1:4. Then, 10 μl of this suspension was inoculated in 8 wells in a 96-well plate with cells at 70% of confluence in the case of established cell lines Vero E6, and with 300,000 cells/well of mature leukocytes (final volume per well: 100 μl). In the primary culture assay, a preparation of 1% homologous red blood cells in buffered saline was also required for hemadsorption formation. In these 8 wells, the surfactant liquid concentration was 1:40 (see Results section). In the same plate, 8 wells were inoculated with the inoculum at the same concentration (2.5x10^5^TCID_50_/ml) without the surfactant liquid (virus control wells), and 8 wells were inoculated with the surfactant liquid at a final concentration of 1:40 (surfactant liquid control wells), while an additional 8 wells were used as cell controls. The plate was incubated for 5 days at 37°C with an atmosphere of 5% CO_2_. Viral viability was evaluated by daily visualization of cytopathic effect (CPE) and cellular death for 5 days, as compared with the controls. Additionally, on the fifth day, the cell cultures were frozen and thawed and the supernatants were collected to perform a molecular diagnosis of each pathogen.

To ensure the virus inactivation in primary leukocytes culture and Vero-E6, the supernatants were subjected to three passes by adding 10 μl/well of the surfactant liquid with the viruses, virus control, cytotoxicity control and, cells control to fresh cells. In the case of leukocytes, 1% homologous red blood cells were also added to the culture.

### Evaluation of SARS-CoV-2 and ASF virus genome stability preservation in the surfactant liquid

For this purpose, an inoculum of 10^6^TCID_50_/ml of both viruses was used. For both viruses, the inoculum was mixed with the surfactant liquid to achieve a final concentration of the virus of 4.4x10^5^TCID_50_/ml. This suspension was aliquoted into several sterile tubes that were stored at different temperature conditions: -20°C, 4°C, room temperature, and 37°C. The different stored tubes (three for each condition and time) for each temperature condition were collected at the following time points: 24 hours, 72 hours, 6 days, 10 days, 15 days, and one month.

The genome stability of both viruses was evaluated at each temperature at each time point using quantitative PCR (qPCR) protocols. In both cases, RNA/DNA was extracted using the column-based High Pure Viral Nucleic Acid Kit (Roche, Basel, Switzerland), according to the manufacturer’s instructions. Total RNA/DNA was suspended in RNase/DNase-free water and used immediately for molecular diagnosis. In the case of the SARS-CoV-2 virus, RNA detection was performed using the envelope protein (E)-encoding gene (Sarbeco) and two targets (IP2 and IP4) of the RNA-dependent RNA polymerase gene (RdRp) in an RT-qPCR protocol established by the WHO following its guidelines (https://www.who.int/emergencies/diseases/novel-coronavirus-2019/technical-guidance/laboratory-guidance) [1]. For the ASF virus, the DNA was amplified by employing the Universal Probe Library (UPL) real-time PCR protocol [27]. The results were expressed in Ct (Cycle threshold) and were considered positive when the Ct value was < 40.0.

### Environmental detection of SARS-CoV-2 and ASF virus

To evaluate the efficacy of the method for environmental sampling, testing of both viruses on potentially contaminated surfaces was conducted during two independent in vivo experiments.

For SARS-CoV-2 environmental evaluation, environmental samples were collected three times per week during an infection experiment with cats, previously described in [28]. The prehydrated sponges were used for recovering viral RNA from the animals´ hair from two cats and the isolator where the animals were housed (including the feeders, water containers, and flor) during the three weeks the experiment lasted.

For ASFv, environmental samples were collected three times per week during an infection experiment with wild boar (data not published). In this experiment, six wild boars were orally inoculated with the naturally attenuated strain Lv17/WB/Rie1 [29]. Environmental samples were collected three times per week for four weeks. Samples included the floor, trough, feeder, facilities and animal skin.

## Results

### Cell cytotoxicity of the surfactant liquid in Vero E6 cells

Cell cytotoxicity of the surfactant liquid was evaluated by measuring cell viability in the presence of different dilutions of the liquid. According to cell visualization and MTT results, Vero E-6 and leukocyte cell growth and viability were adequated when the surfactant liquid was diluted at 1:40. The optical density (OD) in these wells was similar to the OD obtained in the cell control wells (M-W U test, p > 0.05).

Thus, the dilution factor of 1:40 was used for further experiments.

### Viral inactivation capacity of the surfactant liquid during cell culture of SARS-CoV-2 and ASF virus

In order to evaluate if the surfactant liquid can inactivate SARS-CoV-2 and ASF virus, inoculums of these viruses were previously inactivated with the liquid for 5 minutes. Then, the inactivated inoculums were inoculated in susceptible cells. The viral activity was evaluated by visualization of the CPE and qPCR in all the cases, and by visualization of the presence/absence of hemadsorptions in the experiment using leukocyte primary culture.

**Fig 1:**
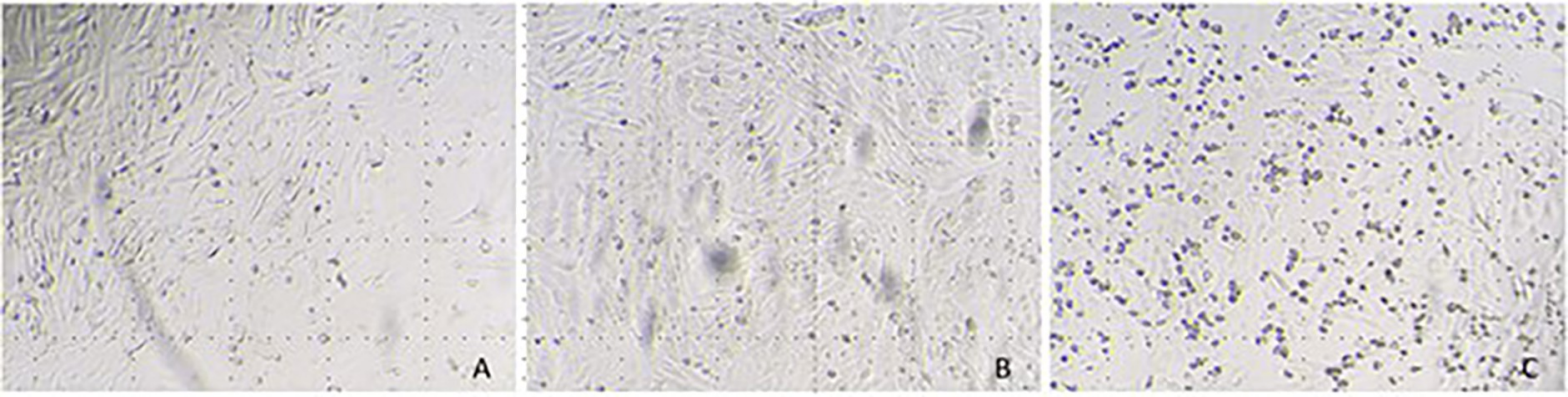
Vero E6 cells inoculated with the inactivated inoculum of SARS-CoV-2 (A), control cells (B), and virus control cells (C) with evidence of the cytopathic effect and cell death.

**Fig 2:**
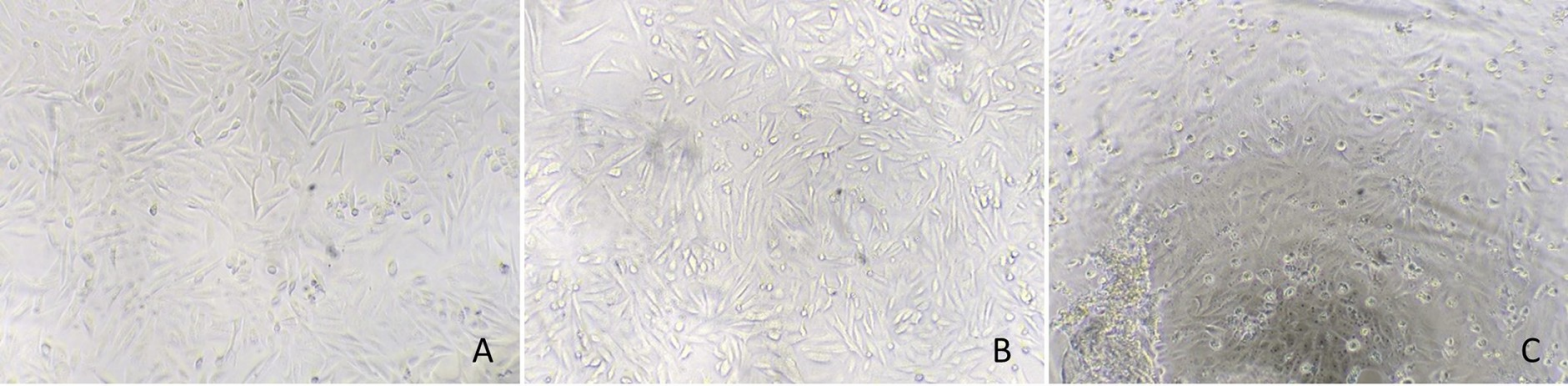
Vero E6 cells inoculated with the inactivated inoculum of African swine fever virus (España-70 isolate) (A), control cells (B), and virus control cells (C) with evidence of the cytopathic effect and cell death.

**Fig 3:**
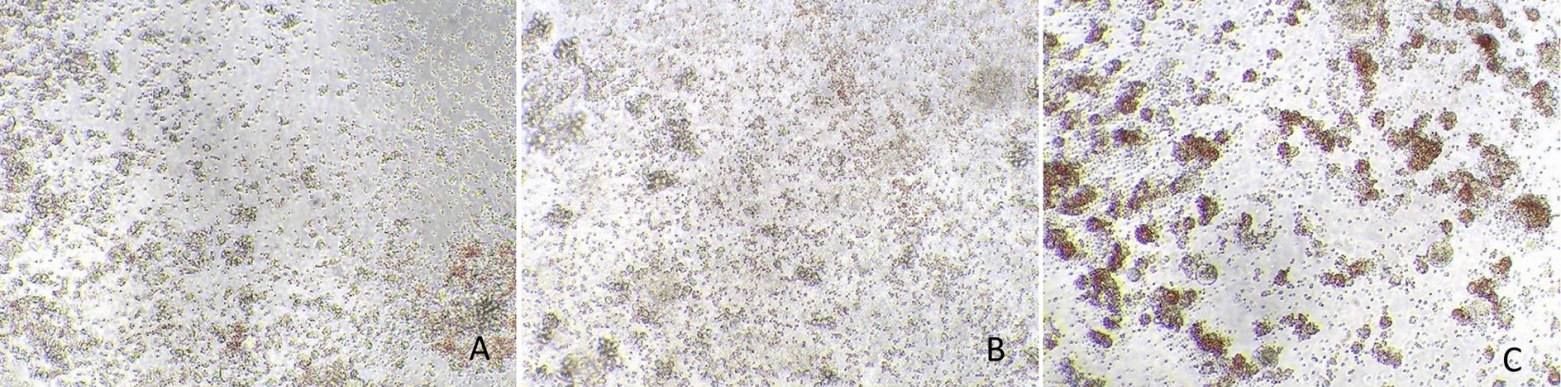
Leukocyte cells inoculated with the inactivated inoculum of African swine fever virus Armenia 07 insolate (A), control cells (B), and virus control cells (C) with evidence of hemadsorption and cytopathic effect.

In the case of the SARS-CoV-2 virus, cells inoculated with the inactivated inoculum presented a high confluence at day 3 post-infection, without cell mortality nor alteration of the cell morphology, similar to those wells of cell controls. In contrast, cells inoculated with the virus control (without the surfactant liquid) presented high CPE and cell death (Fig 1). The results of virus isolation obtained in RT-qPCR confirmed inactivating properties of the surfactant liquid (Table 1). The same occurred in the case of the ASF virus (Figs 2 and 3).

**Table 1:** qPCR Ct (Cycle threshold) values from supernatants collected from the two different cell culture experiments. The virus load was expressed in Ct values obtained by quantitative PCR.

Ct values from SARS-CoV-2 virus isolation experiment in Vero E6 cell culture			
Virus used for inoculation	Mix virus/surfactant liquid	Virus control	Cell control
17.96	24.21	13.44	Negative
Ct values from E70 isolate of African swine fever virus isolation experiment in Vero E6 cell culture			
Virus used for inoculation	Mix virus/surfactant liquid	Virus control	Cell control
20.74	27.89	17.89	Negative
Ct values from Arm07 isolate of African swine fever virus isolation experiment in leukocytes cell culture			
Virus used for inoculation	Mix virus/surfactant liquid	Virus control	Cell control
18.75	26.07	15.12	Negative

### Preservation of the genome of SARS-CoV-2 and ASF virus in the surfactant liquid at different temperature conditions

To evaluate the preservation capacity of the surfactant liquid in both viruses, different temperature conditions, and time points were established. Using qPCR, we detected a high rate of genome conservation in both viruses. In both cases, positive results for qPCR were obtained in all the temperature conditions and at all the time points, although viral loads were more stable in the case of ASF virus.

**Fig 4:**
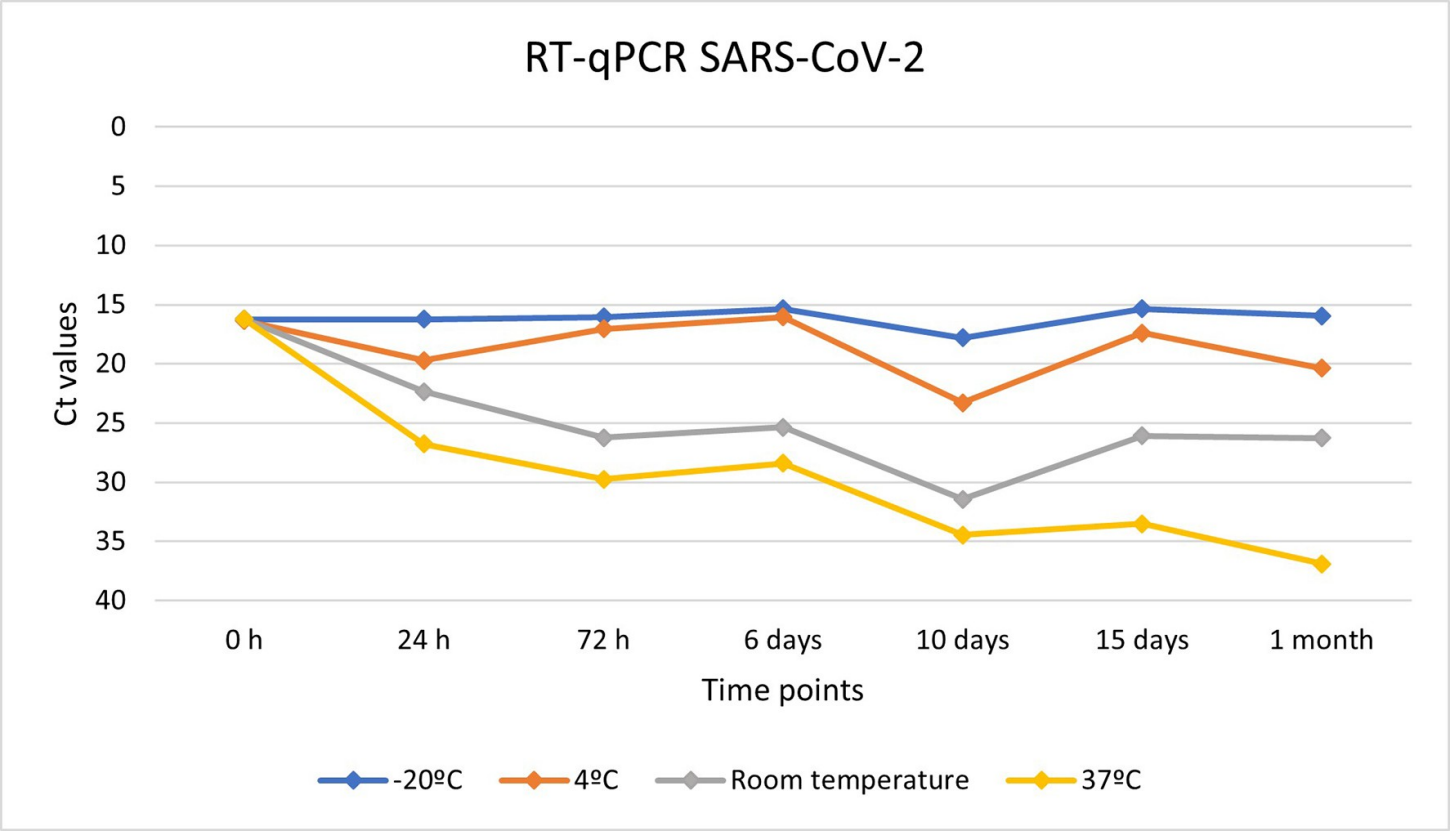
Evolution of the viral loads of SARS-CoV-2 genome preserved in the surfactant liquid based on Ct values at different temperature conditions at different time points.

**Fig 5:**
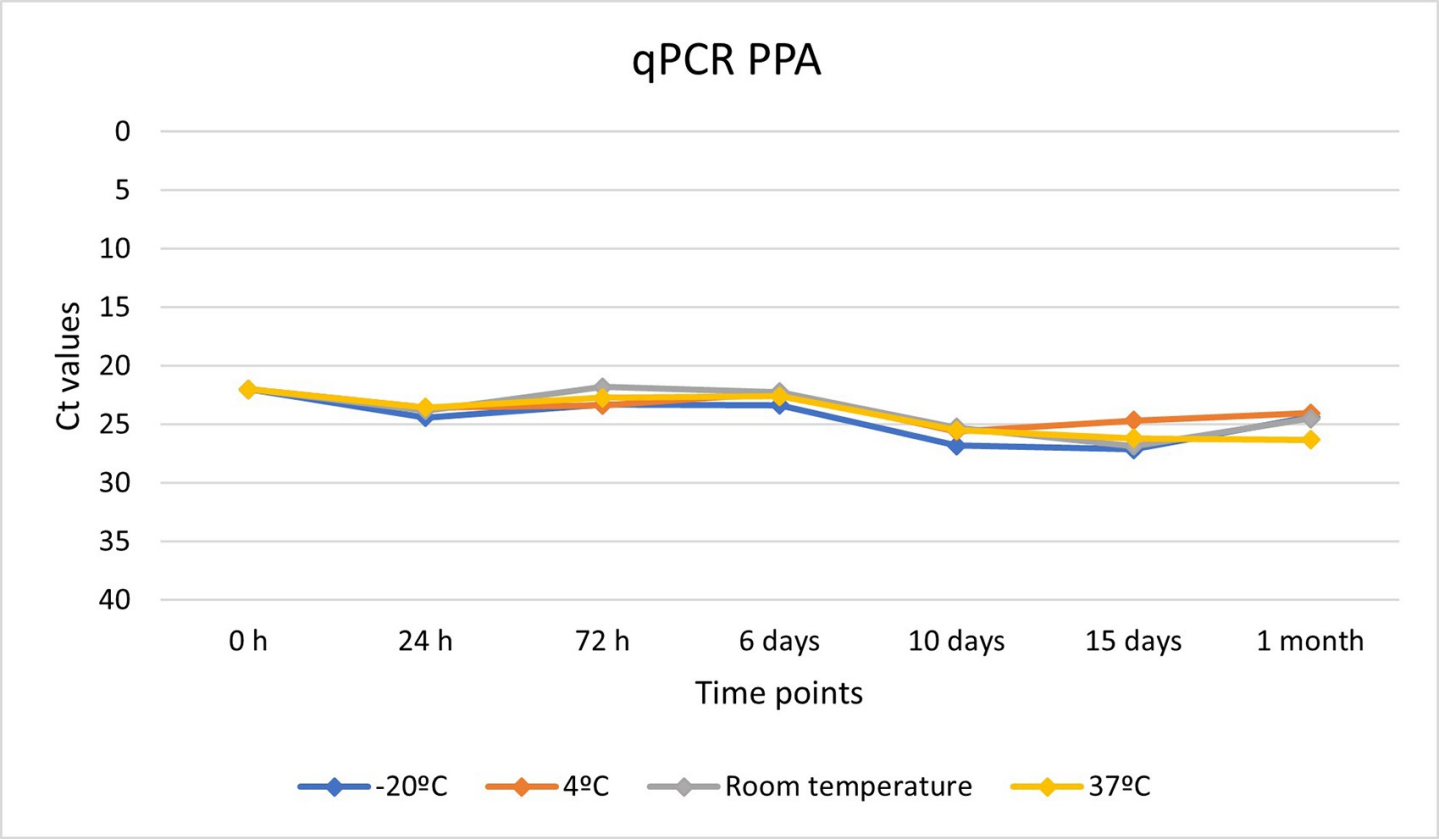
Evolution of the viral loads of the ASF virus genome preserved in the surfactant liquid based on Ct values at different temperature conditions at different time points.

Figs 4 and 5 show the evolution of the viral loads for both viruses at different temperature conditions and at different time points.

### Environmental detection of SARS-CoV-2 and ASF virus during in vivo experiments

**Fig 6:**
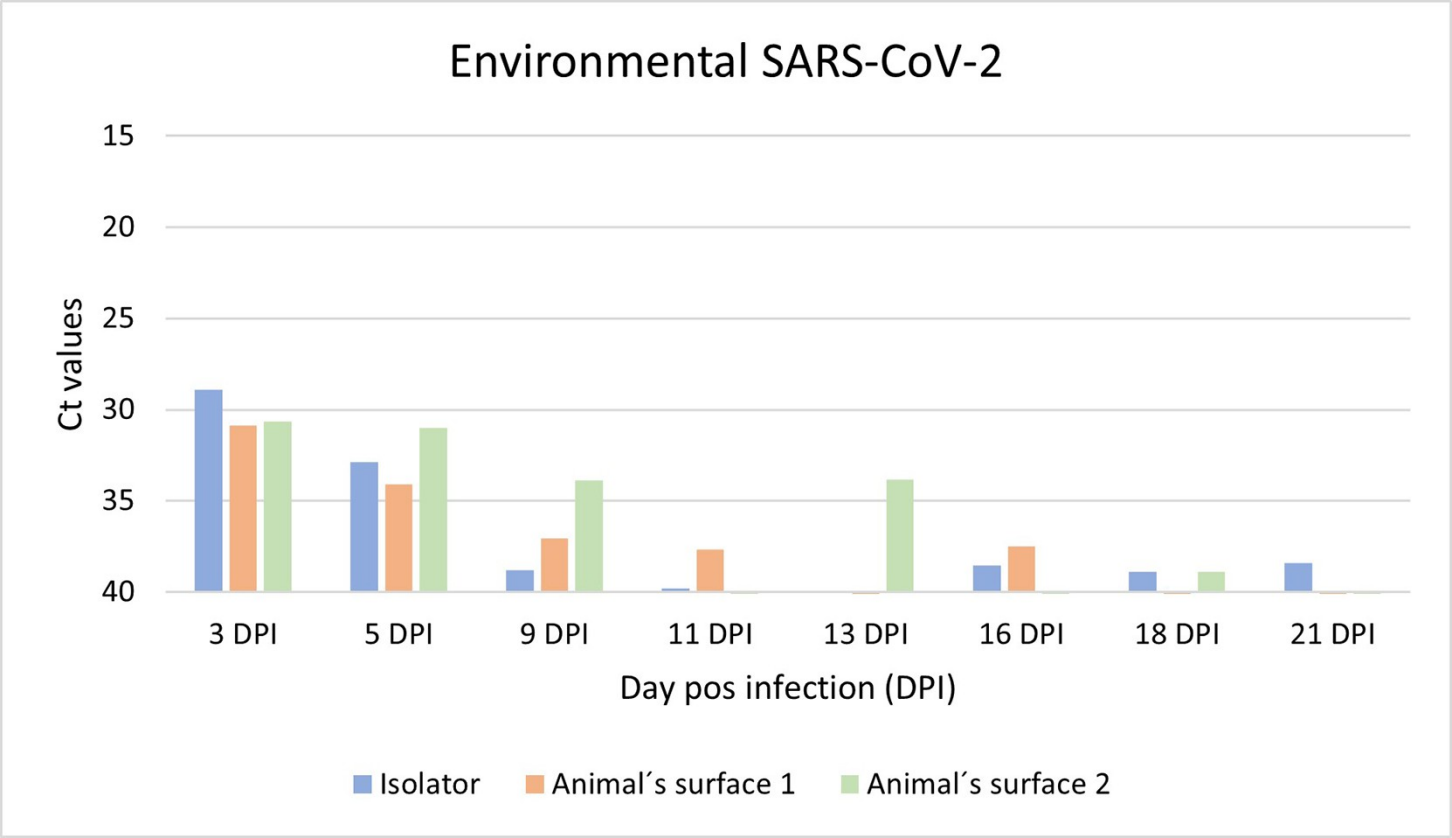
Evolution of Ct values of SARS-CoV-2 genome recovered from the environment in an experimental infection of cats.

SARS-CoV-2 RNA was successfully recovered from the environment during the infection experiment. Results are shown in Fig 6.

**Fig 7:**
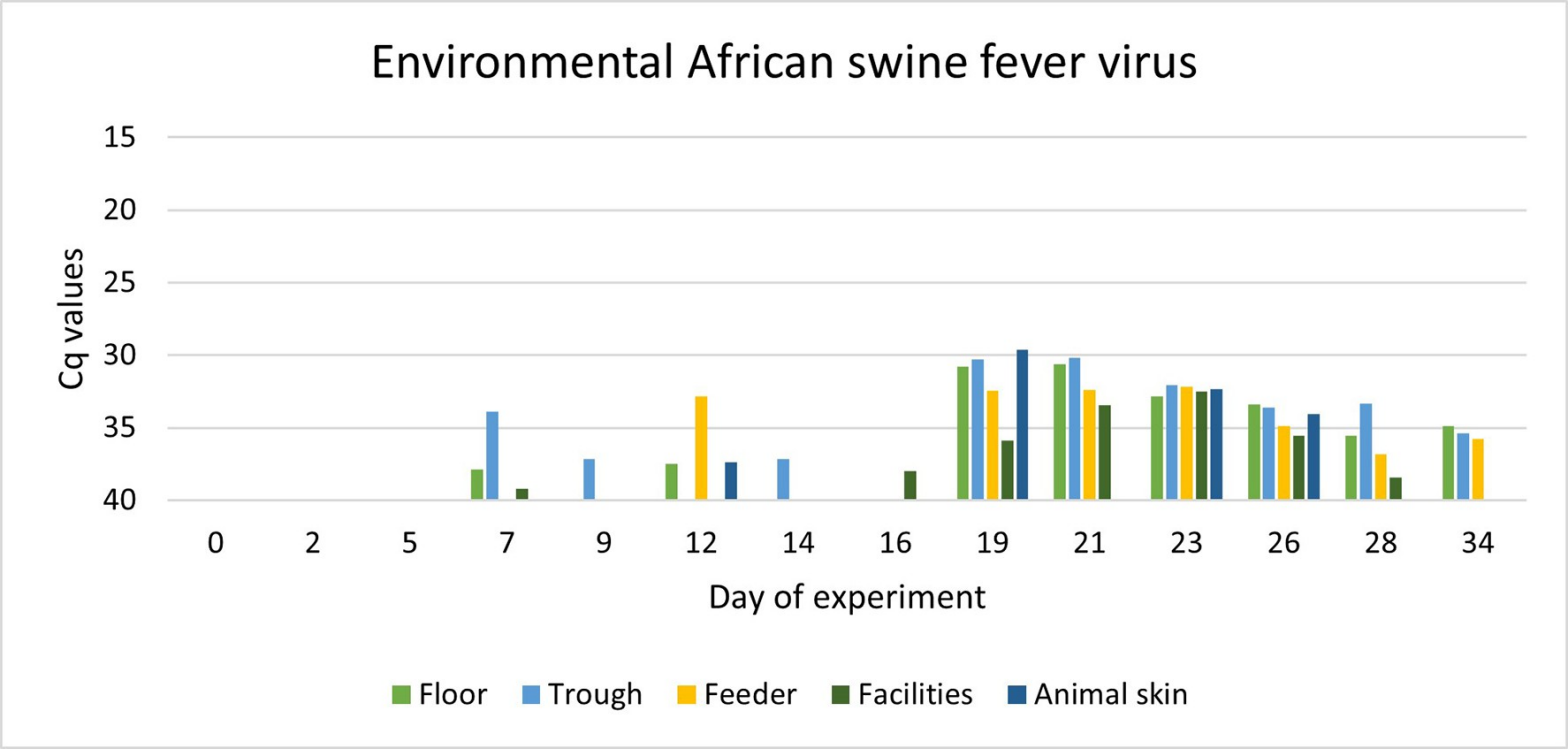
Evolution of Ct values of African swine fever genome recovered from the environment in an experimental infection of wild boar with the naturally attenuated strain Lv17/WB/Rie1.

The same occurred in the case of ASF DNA, which was effectively detected in the environment using the surfactant liquid (Fig 7).

## Discussion

Both SARS-CoV-2 and ASF viruses are currently major concerns in public and animal health. While COVID-19 disease has produced more than 6 million deaths and more than 500 million cases, causing a dramatic economic and social disruption, the spread of ASF disease over the world has led to devastating consequences in the pig industry. Although vaccination seems like the most promising tool for controlling both diseases, preventive measures should be also conducted in order to avoid their transmission. In this sense, a rapid and early diagnosis is critical for applying adequate containment actions. In the case of COVID-19, once the positive case is identified, isolation and the use of masks are helpful to limit virus transmission [30]. On the contrary, severe measures should be applied when an ASF outbreak is detected, which include the depopulation of affected farms [31]. In both scenarios, environmental detection of the agent may be a key point for controlling the diseases, since host sampling is not always possible. This is particularly interesting in the case of ASF disease: for example, the detection of ASF virus genome in trucks destined for pig transport or on surfaces from backyard farms would be a strong indication that the virus is circulating [14, 32, 33]. In the case of SARS-CoV-2, environmental surveillance may contribute to evaluating shedding dynamics, identifying risk factors, and validating decontamination procedures [15]. It may also contribute to performing COVID-19 surveillance in mink farms, where the introduction of the virus may trigger the apparition of an outbreak [34, 35]. In this species (*Neovison vison*), the infection can generate clinical signs, and, most importantly, adaptation to the host may lead to new variants of the virus [36, 37]. It has been also reported that these animals can act as a source of infection for humans [34], which highlight the importance of monitoring SARS-CoV-2 infection dynamics in places with high population density, like farms. All together will increase, definitively, the diagnostic capacity and, therefore, the control of both diseases.

Here, we tested the inactivation and preservation properties of a surfactant liquid for environmental sampling of both SARS-CoV-2 and ASF viruses. Results from cell culture assays have demonstrated that the surfactant liquid effectively inactivates both viruses since no virus growth was detected in the cells inoculated with the inactivated inoculums. No cytopathic effect or cellular death was observed in these cells at the concentration of surfactant liquid tested. On the contrary, cells inoculated with the same viral inoculum without being inactivated with the surfactant liquid did present an evident cytopathic effect, and hemadsorption effect in the case of leukocyte culture infected with Arm07, which is indicative of virus growth. These results were reinforced by molecular diagnosis using PCR. The required time of contact between the infectious sample and the surfactant liquid to achieve inactivation is only five minutes, which is an advantage for sampling and consecutive transport. If the sample is immediately inactivated, biosecurity measures do not have to be as extreme as in the case of infectious ones in terms of transportation and processing. Although SARS-CoV-2 suspected samples can be handled in a BSL2 laboratory, higher biosecurity measures are recommended if available [38]. On the contrary, ASF-free countries must process suspected samples in a BSL3 laboratory, which limits the diagnostic capacity to a few laboratories with adequate facilities [21]. In both cases, inactivation of the sample accelerates the process and increases the number of laboratories capable of performing qPCR, which substantially enhances the diagnostic capacity of the country.

We also evaluated the capacity of the surfactant liquid for both RNA and DNA preservation. In our laboratory assay, viral inoculums of both viruses were inactivated with the surfactant liquid and distributed into homogeneous samples that were stored at different temperature conditions. Congelation, refrigeration, and room temperature were tested, but also a more extreme temperature condition, 37°C, which is often efficient for viral genome degradation. As expected, congelation and refrigeration of the samples successfully preserved the viral genome of both viruses, maintaining viral loads almost identically to the original inoculum. SARS-CoV-2 RNA showed high stability at these temperature conditions, being the viral loads based on Ct values very similar to the original inoculum despite the passing of time. RNA losses were observed when the samples were subjected to higher temperatures, but the surfactant liquid permit the detection of the RNA even in sample stored at 37°C for one month. These results show the utility of the method for preserving viral RNA, which is easily degraded [39]. Therefore, the use of the surfactant liquid as the sampling methodology significantly helps to increase RNA preservation. This is particularly important for field data collection, where the time between sampling and storing may be a critical point. With the implementation of this new methodology, the problem is solved.

In the case of ASF virus genome, the results were even better: Ct values obtained from the different temperature conditions at the different time points were hardly ever identical to the original inoculum, which demonstrates that the surfactant liquid is a fantastic tool for preserving ASF virus genome in suspected samples. The greater efficiency observed in the case of ASF virus with respect to the SARS-CoV-2 virus can be due to the high stability of its genome, which consists of a double strand of DNA [6]. ASF virus DNA is known to be resistant to environmental conditions for long periods. In fact, the virus remains infectious over a long storage time [6]. This supports the results from this study, where the DNA was perfectly preserved even at 37°C for one month. Therefore, as ASF virus DNA is easier to preserve, the additional use of the surfactant liquid tested here would be an improvement that suspected ASF virus environmental samples may be conserved for a very long period of time.

The surfactant liquid tested in this study has been previously used for environmental samplings of several pathogens, such as *Mycobacteria tuberculosis* [22], SARS-CoV-2 [15], ASF virus [14] and *Brucella suis* [23]. Here, we also observed a good rate of genome recovery for both viruses, as demonstrated by the environmental detection performed during experimental infections. Therefore, this methodology for environmental sampling has shown robust results for several pathogens that prove its efficacy. The complementary laboratory analyses conducted in this paper demonstrate the inactivation and preservation capacities of the surfactant liquid for two different viruses with very different characteristics, which suggests that these properties would be maintained in other pathogens.

In conclusion, this new methodology for environmental sampling may open a door for the indirect detection of relevant pathogens, facilitating sampling, sample transport, and processing and, therefore, increasing animal welfare and the diagnostic capacity of the laboratories and, thus, the control of two important diseases in terms of One Health.
